# Cognitive impairment in syphilis: Does treatment based on cerebrospinal fluid analysis improve outcome?

**DOI:** 10.1371/journal.pone.0254518

**Published:** 2021-07-13

**Authors:** Arielle P. Davis, Clare L. Maxwell, Haley Mendoza, Abby Crooks, Shelia B. Dunaway, Sher Storey, Claire Stevens, Lauren C. Tantalo, Sharon K. Sahi, Kevin R. Robertson, Christina M. Marra

**Affiliations:** 1 Department of Neurology, Division of Infectious Diseases, University of Washington, Seattle, Washington, United States of America; 2 Division of Infectious Diseases, Department of Medicine, University of Washington, Seattle, Washington, United States of America; 3 Department of Neurology, University of North Carolina, Chapel Hill, North Carolina, United States of America; Lluita contra la SIDA Foundation - Germans Trias i Pujol University Hospital, SPAIN

## Abstract

**Background:**

Individuals with previous syphilis may experience cognitive impairment. The goal of this study was to determine if those at high risk for laboratory-defined neurosyphilis are cognitively impaired, and whether treatment based on cerebrospinal fluid (CSF) findings results in better outcomes.

**Methods:**

Participants had a new syphilis diagnosis, serum RPR titer ≥ 1:32 or peripheral blood CD4+ T cells ≤ 350/ul (in persons living with HIV) and did not endorse neurological symptoms. They underwent computerized cognitive assessment with the CogState. Thirty-two were randomized to either undergo lumbar puncture (LP) or to not undergo LP and 14 underwent LP; 64 were not randomized and 48 opted to undergo LP.

**Results:**

Demographics, cognitive complaints and cognitive impairment did not differ between randomized and nonrandomized participants. Two-thirds were cognitively impaired, and impairment was not more common in those with cognitive complaints. The adjusted odds of increased severity of impairment were 3.8 times greater in those with CSF pleocytosis compared to those without. Time to cognitive normalization, improvement or decline did not differ between those who did not undergo LP and those who underwent LP and whose treatment was based on CSF analysis. Taking into account pre-treatment cognitive impairment, the risk of cognitive decline was lower in those with CSF pleocytosis treated for neurosyphilis compared to those without CSF pleocytosis not treated for neurosyphilis, (HR 0.24 (95% CI 0.07–0.88], p = 0.03).

**Conclusion:**

In individuals at high risk for laboratory-defined neurosyphilis, cognitive complaints are not a good indicator of cognitive impairment. Severity of cognitive impairment was greater in those with CSF pleocytosis. Identification and treatment of those with neurosyphilis may mitigate subsequent cognitive decline.

## Introduction

Lumbar puncture (LP) was routine in patients with syphilis in the pre-antibiotic era but seemed unnecessary after introduction of penicillin in the mid 1940s. However, in the early 1980s, reports of neurosyphilis in persons living with HIV (PLWH) after appropriate early syphilis treatment, termed “neurorelapse,” led to recommendations to perform LPs to identify asymptomatic neurosyphilis. The 2002 Centers for Disease Control and Prevention (CDC) guidelines recommended LP for all PLWH with late latent syphilis or syphilis of unknown duration [1]. In response to reports showing that cerebrospinal fluid (CSF) abnormalities were more common in individuals with syphilis and serum rapid plasma reagin (RPR) titers ≥1:32, or, in PLWH with peripheral blood CD4+ T cells ≤350/ul, the 2006 guidelines suggested consideration of LP in individuals who met these criteria [2]. Current European guidelines suggest considering CSF examination in asymptomatic PLWH with late syphilis and CD4+ T cells ≤350/ul or a serum Venereal Disease Research Laboratory (VDRL) or RPR titer >1:32 [3]. However, CDC guidelines since 2010 recommend against LP unless neurologic symptoms are present, stating, without citation, that CSF examination in absence of neurologic symptoms is not associated with improved clinical outcomes [4, 5]. Based on uncertainty regarding which patients with syphilis should undergo LP, and data suggesting that PLWH with prior syphilis suffer cognitive sequelae [6, 7], we undertook a study to address whether individuals with syphilis who underwent LP had a better outcome than those who did not. The hypotheses to be tested were 1) individuals with higher serum RPR titers, or lower peripheral blood CD4+ T cells in PLWH, who do not meet current CDC criteria for LP, have cognitive and functional abnormalities on formal testing; and 2) LP in these individuals, with treatment based on CSF results, results in better cognitive and functional outcomes. We report here the results of analyses testing both hypotheses.

## Methods

The University of Washington Institutional Review Board approved this study, via written consent (IRB approval numbers: STUDY00001718, STUDY00003216). Participants were enrolled from September 2013 to March 2018. Study enrollment criteria changed over its course because of slow accrual. The initial enrollees were PLWH with a new syphilis diagnosis who had serum RPR titer ≥ 1:32 or peripheral blood CD4+ T cells ≤ 350/ul who did not meet CDC criteria for lumbar puncture based on symptoms [5]. These participants were randomized to LP vs. no LP. After the first year only 32 randomized individuals were entered, and the study was opened to persons not infected with HIV and to individuals who declined randomization. The subsequent 187 individuals were not restricted by serum RPR titer, peripheral blood CD4 or symptoms. These enrollees were then screened and included in the analyses if they met the eligibility criteria for the original study. The distinction between the initial enrollees was that the decision to undergo LP was randomized, whereas for the subsequent enrollees, the decision was made by the participant, his or her health care provider, or both. We systematically collected the reason for not agreeing to randomization in 168 participants. The most common was that the provider recommended LP (n = 133). Individuals who were randomized were not allowed to re-enroll in the study with a new episode of syphilis, but those who were not randomized could re-enter and opt to be randomized or not with a subsequent episode of syphilis.

**Fig 1:**
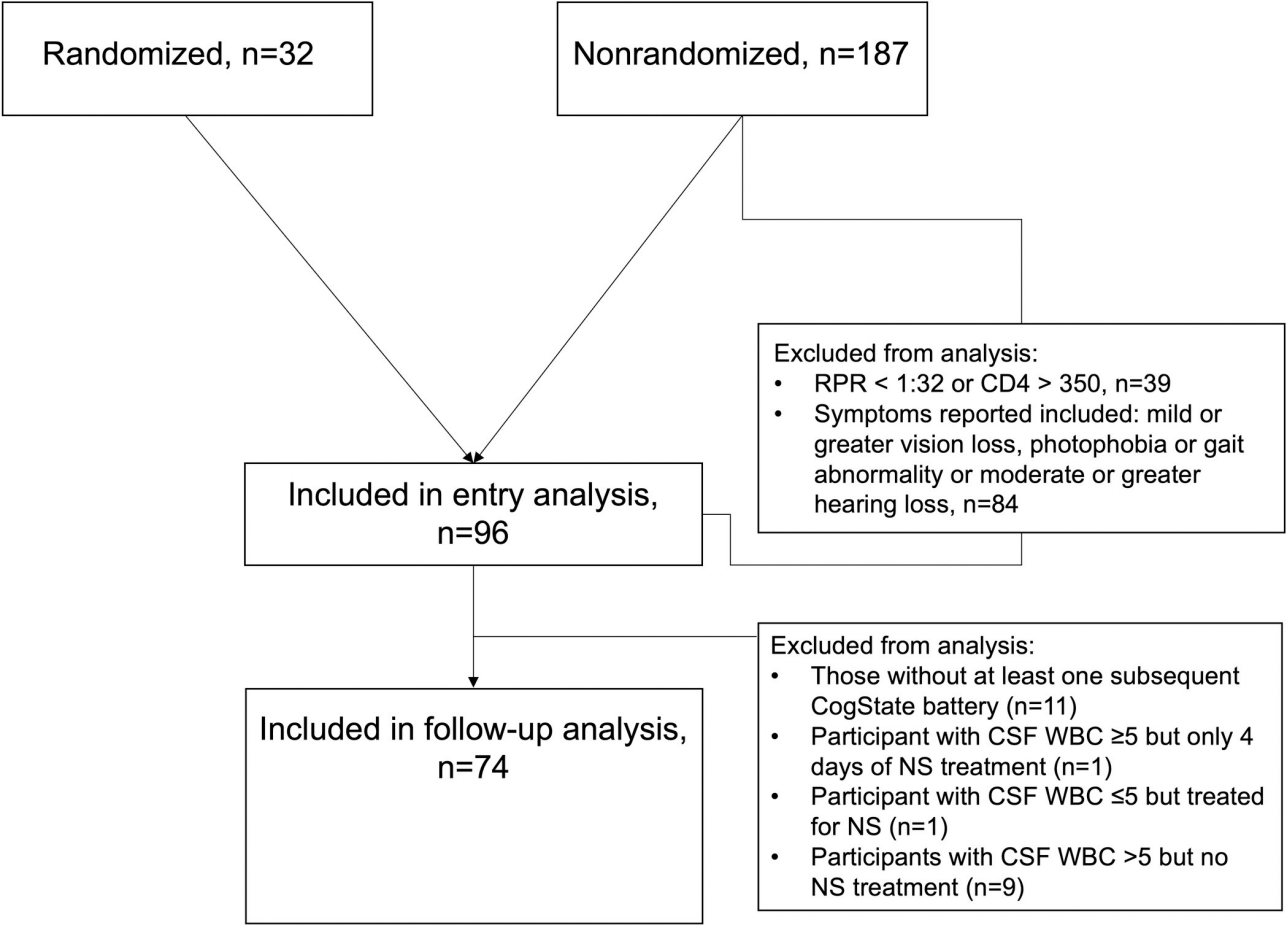
Flow diagram of participants included in entry and follow-up analyses. RPR, Rapid Plasma Reagin.

The first randomized visit and the first nonrandomized visit for those who were never randomized were included in the entry analysis if the participant completed the CogState battery. All randomized participants were included in the analysis; 2 individuals were randomized after a previous, nonrandomized syphilis episode, 6 and 9 months later. Of the 187 nonrandomized participants, analysis was restricted to those meeting the original entry criteria of serum RPR titer ≥ 1:32 or, for PLWH, peripheral blood CD4+ T cells ≤ 350/ul. All nonrandomized participants endorsed normal vision, no photophobia or gait abnormality, and normal or at most mild hearing loss based on our previous observation that these symptoms predicted a reactive CSF-VDRL [8]. Ninety-six individuals were included in the entry analysis (Fig 1). Follow-up analysis was restricted to individuals who underwent at least one follow-up CogState assessment, and either did not have an LP or had an LP and had their treatment based on CSF findings. Seventy-four individuals were included in the follow-up analysis (Fig 1).

### Procedures

Participants underwent a medical history, neurologic examination, blood draw, urine toxicology screen, and lumbar puncture, when indicated. The participant’s health care provider and the participant together decided whether neurosyphilis treatment was indicated based on CSF abnormalities alone; neither were aware of cognitive assessment results. Participants who did not undergo lumbar puncture were treated for uncomplicated syphilis according to CDC guidelines [4, 5].

#### Neuropsychological assessment

The neuropsychological assessment included the Wide Range Achievement Test-third edition (WRAT-3) reading subtest [9] as an assessment of premorbid verbal intelligence, a revised version of the Lawton and Brody instrumental activities of daily living (IADL) scale [10, 11] and the Medication Management Test-Revised (MMT-R) [10, 12], the Beck Depression Inventory (BDI-II) [13], and the CogState battery [14]. The CogState consists of computerized neuropsychological tests and has been used in clinical and research settings, including testing in PLWH [15–18]. It includes seven tests spanning cognitive domains of psychomotor function, attention, working memory, executive function, and verbal learning. To assess subjective cognitive impairment, we used question 19 on the IADL (difficulties due to primary cognitive problems or to cognitive and physical problems) and question 19 on the BDI-II (difficulty with concentration). Participants were asked to return at 24 and 52 weeks for follow-up IADL, MMT-R and CogState battery.

#### Laboratory methods

Cerebrospinal fluid white blood cell (WBC) enumeration and CSF-VDRL reactivity were determined in a Clinical Laboratory Improvement Amendments (CLIA)-certified hospital clinical laboratory. HIV RNA and peripheral blood CD4+ T lymphocyte concentrations were obtained by medical record review. Serum RPR titers were performed in a research laboratory [19]. Detection of *Treponema pallidum* subspecies *pallidum* (*T*. *pallidum*) DNA in blood was determined by polymerase chain reaction (PCR) as previously described [20, 21]. Only samples collected within 8 days of treatment of the current episode of syphilis were tested based on our experience that *T*. *pallidum* DNA is rarely detected in blood samples after this time (unpublished data).

#### Data analysis

Assessment of cognitive impairment was based on age adjusted normative data from CogState and was categorized as none (all test scores > -1 standard deviation [SD] of normative data), mild impairment (two test scores < -1 SD, or one test score < -2 SD), moderate impairment (two test scores < -2 SD) or severe impairment (three test scores < -2 SD). For the entry analysis, the latter two categories comprised few participants and were combined. For the follow-up analysis, participants with any impairment at study entry were categorized as improved if they improved by at least one category, or as normalized if they improved to no impairment. Participants not severely impaired at entry were categorized as having declined if they declined by at least one category.

For this study, we defined abnormal CSF as WBCs >5/ul. Because we included persons not infected with HIV and because our PLWH were largely taking antiretrovirals with suppressed plasma HIV RNA, we were unlikely to overdiagnose neurosyphilis.

WRAT-3 scores were normalized based on age [9] and categorized as ≤ 86 vs > 86, the median WRAT-3 of the participants in the entry visit analyses. Years of education was categorized as 9–12 vs. ≥13 years (high school or less vs. more than high school), BDI- II was categorized as 0–13 (none or mild depression) vs. ≥14 (moderate or greater depression), MMT-R was categorized as <5 vs. ≥5 [10, 22], and IADL was categorized as dependent vs. independent.

Descriptive statistics are expressed as number (percent) or median (interquartile range [IQR] or 95% confidence intervals). Proportions were compared using the Chi-square or Fisher’s exact test, and comparisons of continuous variables using Mann-Whitney U test. Odds ratios (ORs) with 95% CI for categories of cognitive impairment at study entry were determined using univariate or multivariate ordinal logistic regression. Time to cognitive change was assessed using the log rank test, and hazard ratios were determined using Cox regression. P-values <0.05 were considered statistically significant.

## Results

### Cognitive impairment at study entry

Of the participants included in the entry analyses, 32 were randomized and 64 were not (Fig 1). They were primarily white men living with HIV, in their mid-30s, similar to the characteristics of patients with syphilis in Seattle King County during the time that the study was conducted [23] (Table 1). As expected because of later, more inclusive entry criteria, more PLWH were in the randomized compared to the non-randomized group. Median serum RPR titer was 128. Three quarters of participants had been treated for the current episode of syphilis, a median of 6 (IQR 0–8) days before study entry in the randomized group, and 5 (IQR 1–16) days in the non-randomized group (p = 0.19). In the randomized group, 43.8% underwent LP, and in the non-randomized group, 75.0% of participants underwent LP; LP could not be performed due to technical reasons in one individual randomized to the procedure. Cerebrospinal fluid VDRL was reactive in 6.5%, and pleocytosis (CSF WBCs >5/μl) was present in 48.4%; all four individuals with reactive CSF-VDRL also had CSF WBCs >5/ul. No participant was treated for neurosyphilis without undergoing LP, and the proportion of individuals who underwent LP and were treated for neurosyphilis did not differ between the randomized and nonrandomized participants (5 [35.7%] of 14 vs. 17 [35.4%] of 48, p = 1.0).

**Table 1:** Demographic and syphilis-related characteristics at study entry.

	All, n = 96	Randomized, n = 32	Not Randomized, n = 64	P-value
Male	95 (99.0)	31 (96.9)	64 (100.0)	0.33
White	71 (74.0)	25 (78.1)	46 (71.9)	0.12
Black	10 (10.4)	5 (15.6)	5 (7.8)	
Other	15 (15.6)	2 (6.3)	13 (20.3)	
Age	35 (28–45)	35 (28–46)	34 (28–45)	0.55
HIV	64 (66.7)	26 (81.3)	38 (59.4)	0.03
Current ARV use	56 (90.3) (n = 62)	22 (88.0) (n = 25)	34 (91.9) (n = 37)	0.68
CD4 count	540 (285–733) (n = 64)	500 (273–766) (n = 26)	573 (287–703) (n = 38)	0.89
HIV RNA	40 (40–1424) (n = 64)	40 (40–809) (n = 26)	40 (40–2232) (n = 38)	0.40
Early syphilis	82 (85.4)	27 (84.4)	55 (85.9)	1.0
Serum RPR titer	128 (64–256)	128 (64–256)	128 (32–256)	0.22
Detectable *T*. *pallidum* DNA in blood	9 (14.1) (n = 64)	3 (12.5) (n = 24)	6 (15.0) (n = 40)	1.0
Treated for current episode of syphilis before entry	73 (76.0)	24 (75.0)	49 (76.6)	0.87
Prior syphilis	33 (34.4)	15 (46.9)	18 (28.1)	0.07
Underwent LP	62 (64.6)	14 (43.8)	48 (75.0)	0.003
Reactive CSF-VDRL	4 (6.5)	1 (7.1)	3 (6.3)	1.0
CSF WBCs>5/ul	30 (48.4)	9 (64.3)	21 (43.8)	0.23

Abbreviations: HIV, human immunodeficiency virus; ARV, antiretroviral; RPR, rapid plasma reagin; LP, lumbar puncture; CSF, cerebrospinal fluid; VDRL, venereal disease research laboratory; WBC, white blood cell.

Cognitive complaints were common in the randomized and nonrandomized groups, and the proportion did not significantly differ between them (Table 2). Cognitive complaints based on either IADL or BDI-II were not more common in those whose urine toxicology screen showed either amphetamine/methamphetamine or THC (12 [52.2%] of 23 positive vs. 34 [47.9%] of 71 negative for amphetamine/methamphetamine had cognitive complaints (p = 0.72), and 10 [45.5%] of 22 positive vs. 36 [50.0%] of 72 negative for THC had cognitive complaints (p = 0.71)). However, cognitive complaints based on either IADL or BDI-II were more common in PLWH compared to those without HIV (36 [57.1%] of 63 vs. 10 [32.3%] of 31, p = 0.02). Similarly, cognitive complaints based on the IADL were more common in those with moderate or greater depression compared to those with mild or no depression (17 [54.8%] of 31 vs. 11 [17.5%] of 63 P<0.001).

**Table 2:** Cognitive, functional and related factors at study entry.

	All, n = 96	Randomized, n = 32	Not Randomized, n = 64	P-value
Cognitive complaints by:				
• IADL	28 (29.8) (n = 94)	11 (35.5) (n = 31)	17 (27.0) (n = 63)	0.40
• BDI-II	38 (39.6)	14 (43.8)	24 (37.5)	0.56
• Either IADL or BDI-II	46 (48.9) (n = 94)	17 (54.8) (n = 31)	29 (46.0) (n = 63)	0.42
Education ≤12 yrs.	23 (24.0)	6 (18.8)	17 (26.6)	0.40
Normalized WRAT-3	87 (83–90) (n = 94)	86 (81–90)	88 (84–90) (n = 62)	0.09
Cognitive				0.50
Impairment				
None	34 (35.4)	12 (37.5)	22 (34.4)	
Mild	37 (38.5)	14 (43.8)	23 (35.9)	
≥Moderate	25 (26.0)	6 (18.8)	19 (29.7)	
MMT-R ≤ 5	5 (5.2)	3 (9.4)	2 (3.1)	0.33
IADL dependent	4 (4.4) (n = 91)	2 (6.5) (n = 31)	2 (3.3) (n = 60)	0.60
BDI-II total score ≥14	31 (32.3)	10 (31.3)	21 (32.8)	0.88
Urine amphetamine or methamphetamine	24 (25.0)	8 (25.0)	16 (25.0)	1.0
Urine THC	23 (24.0)	4 (12.5)	19 (29.7)	0.08

Abbreviations: IADL, instrumental activities of daily living; BDI-II, Beck Depression Inventory; WRAT-3, Wide Range Achievement Test; THC, tetrahydrocannabinol.

Two-thirds of participants scored in the abnormal range on the CogState test battery (Table 2), and again this proportion did not differ between those participants randomized and those not randomized. Among study participants less depressed (BDI-II score of ≤ 13), or with higher achievement test scores (WRAT-3 > 86), or more education (≥ 13 years), the proportion who scored in the impaired range was similar, 52.0–58.9%. In participants with all three attributes (less depression, higher achievement test scores and more education), 12 (40.0%) of 30 scored in the impaired range. There was no significant relationship between cognitive complaints and performance on the CogState battery, CSF pleocytosis or a reactive CSF-VDRL (S1 Table).

**Fig 2:**
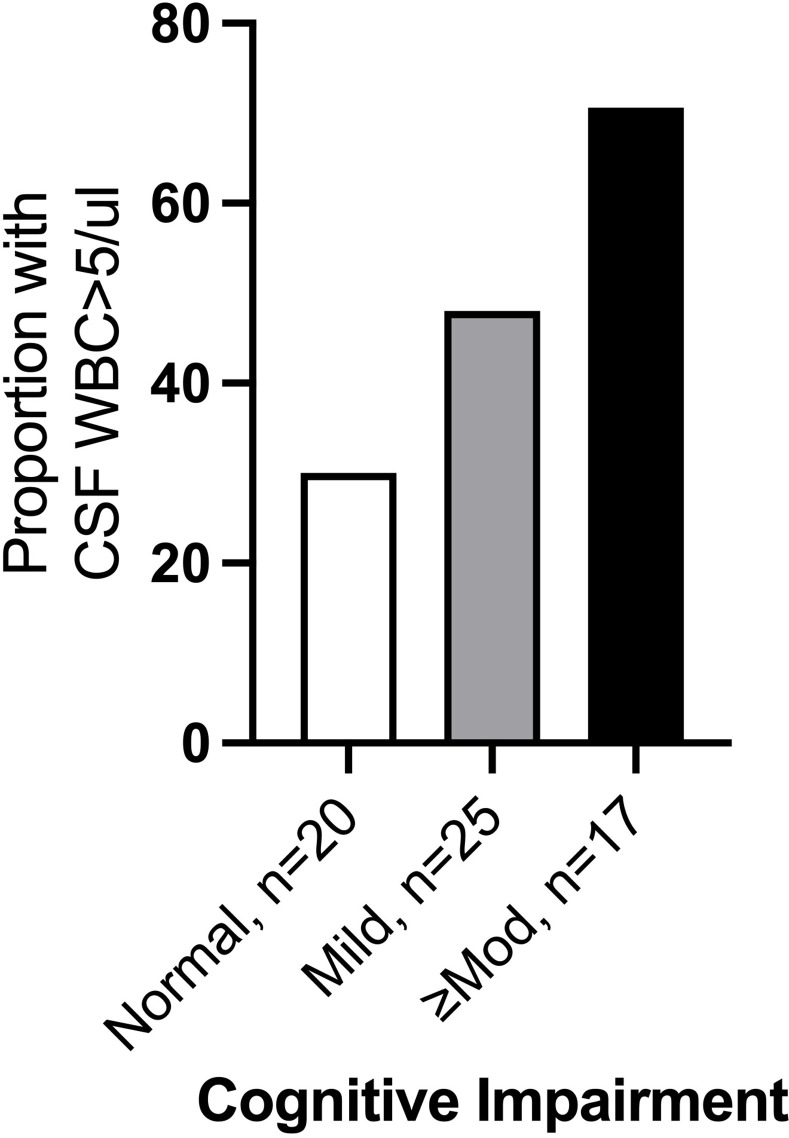
Bar graph demonstrating that severity of cognitive impairment increased as the proportion of participants with Cerebrospinal Fluid (CSF) pleocytosis increased. WBC, white blood cells.

**Fig 3:**
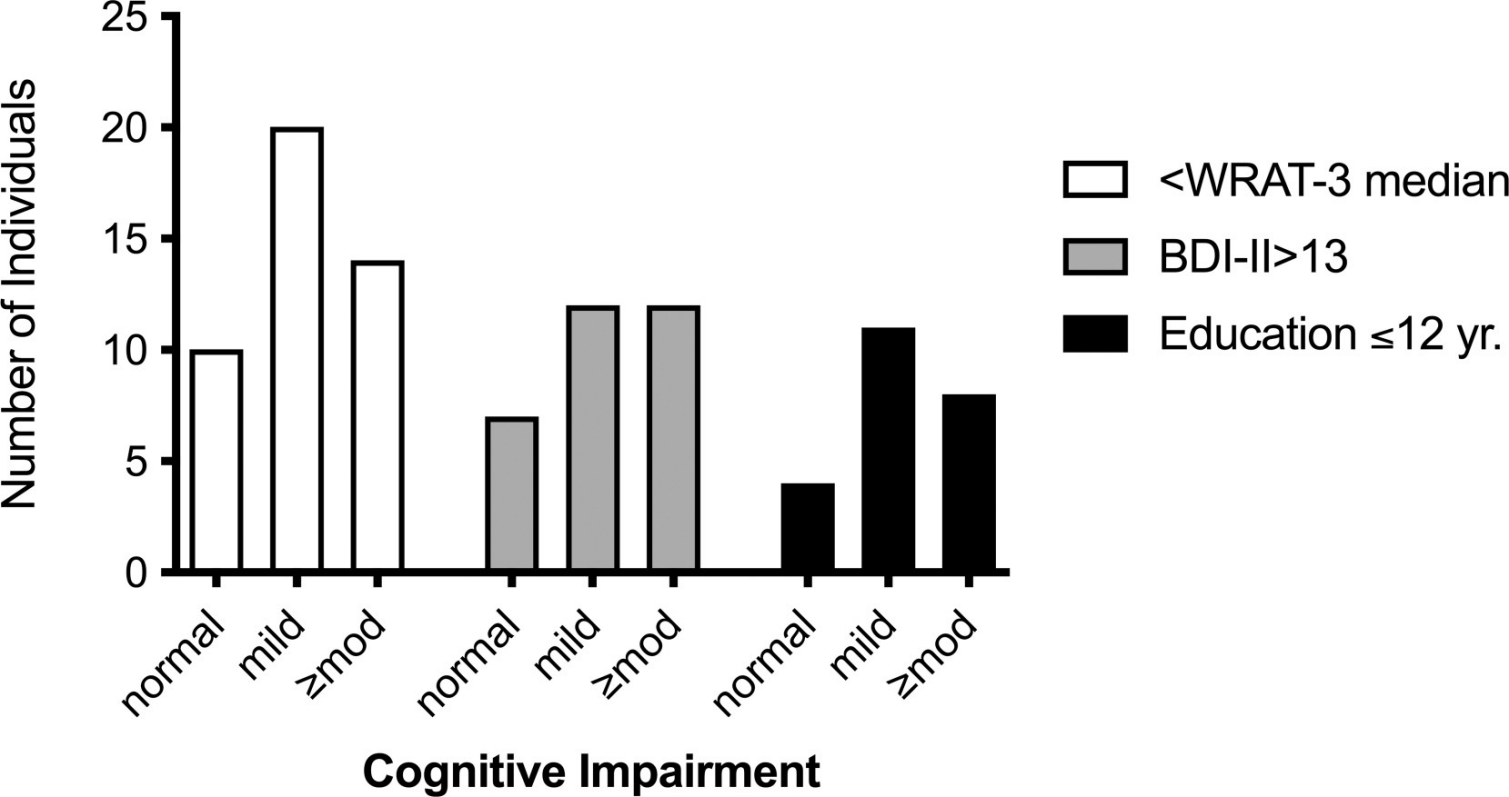
Bar graph of severity of cognitive impairment in those with entry Wide Range Achievement Test–third edition score (WRAT-3) scores below the median, moderate or greater depression by Beck Depression Inventory-II (BDI-II) score at entry and 12-years of education or less.

The severity of cognitive impairment was not related to randomization status, HIV, treatment of the current episode of syphilis prior to entry, serum RPR, syphilis stage, detection of *T*. *pallidum* in blood, prior syphilis or urine toxicology screen positive for either amphetamine and methamphetamine or THC (S2 Table). Severity of cognitive impairment was greater in individuals with CSF pleocytosis (Table 3 and Fig 2), with moderate or greater depression, with less than a high school education (Table 3 and Fig 3), and with WRAT-3 scores below the group median (Table 3 and Fig 3). In multivariate analysis including all four variables, WRAT-3 score was no longer significant. Controlling for the remaining significant variables, the odds of greater severity of cognitive impairment were 3.8 times higher in individuals with CSF pleocytosis (Table 3). Adding randomization status to the final model did not alter the observed relationships (S3 Table).

**Table 3:** Factors that influence severity of cognitive impairment as assessed by CogState.

	Severity of Cognitive Impairment	
Factor	OR (95% CI), p-value	aOR (95% CI), p-value
CSF WBCs > 5/ul	3.3 (1.3–8. 8), p = 0.02	3.8 (1.4–10.6), p = 0.009
Total BDI-II ≥14	2.5 (1.1–5.6), p = 0.03	5.2 (1.7–15.8), p = 0.004
≤ 12 years of education	2.3 (1.0–5.4), p = 0.06	3.3 ((1.0–10.2), p = 0.04
WRAT-3 ≤ 86	2.5 (1.2–5.4), p = 0.02	--

Abbreviations: CSF, cerebrospinal fluid; BDI-II, Beck Depression Inventory.

### Change in cognition over one year

Seventy-four individuals underwent at least one follow-up CogState assessment and either did not have an LP (n = 32) or had an LP and neurosyphilis treatment based on the results (n = 42) (Fig 1). Of those who had their treatment based on CSF findings: 23 had CSF WBC ≤5/ul and were not treated for neurosyphilis, and 19 had CSF WBCs >5/ul and were treated for neurosyphilis. In the latter group, median CSF WBCs was 18/ul (9–29) and CSF-VDRL was reactive in three. Treatment for neurosyphilis included IV penicillin G or IV ceftriaxone, or IM procaine penicillin G with oral probenecid. All were treated for at least 9 days (median 9 [9–10]).

Compared to those not included in the follow-up analysis, participants in the follow-up analysis were less likely to be PLWH (19 [86.4%] of 22 vs. 45 [60.8%] of 74, p = 0.04), were less likely to have undergone LP at entry (20 [90.9%] of 22 vs. 42 [56.8%] of 74, p = 0.004, and showed a trend towards being more likely to have normal cognition at entry (4 (18.2%) of 22 vs. 30 (40.5%) of 74, p = 0.08). There were no other significant differences between those not included and those included in the follow-up analysis, in particular, no difference in the proportion randomized versus not randomized.

Of the 44 individuals with any initial cognitive impairment, 20 (45.5%) improved in their CogState impairment category and 6 (13.6%) reverted to normal cognition. Of the 67 individuals with initial less than severe impairment, 27 (40.3%) experienced decline in CogState performance. There was no difference in time to cognitive improvement or normalization, or in time to cognitive decline in those who did not have an LP compared to those who had an LP and whose treatment was based on CSF findings (S4 Table).

We examined cognitive decline in detail because of reports of cognitive impairment in individuals with previous syphilis [6, 7, 24]. The hazard ratio for cognitive decline was not influenced by randomization status, syphilis stage, serum RPR titer, HIV status, antiretroviral use or peripheral blood CD4+ T cell concentration in PLWH, cognitive performance before treatment, BDI or WRAT-3 scores. Taking into account pre-treatment cognitive performance (because severity of cognitive abnormalities was greater in individuals with CSF pleocytosis), the risk of cognitive decline was significantly lower in those with CSF pleocytosis treated for neurosyphilis compared to those without CSF pleocytosis not treated for neurosyphilis (HR 0.24 [95% CI 0.07–0.88], p = 0.03). Adding randomization status to the model did not attenuate the observed relationship.

## Discussion

In this group of individuals with syphilis at high risk of CSF-defined neurosyphilis, we found that objective evidence of cognitive impairment was common, with one-third of individuals having mild and one third having moderate or greater impairment. Moreover, the adjusted odds of increasing severity of cognitive impairment were 3.8 times higher in those with CSF WBCs > 5/ul compared to those with ≤ 5/ul. Cognitive impairment was not more common in those with cognitive complaints. Our findings of greater cognitive impairment in those with lower pre-morbid intelligence, lower education and more depression support the validity of our cognitive testing; these associations have been demonstrated in other populations and are well known [25–27].

Cognitive impairment in PLWH with prior syphilis was first documented in 1997 by Wallace and colleagues [7]. An analysis of the CHARTER cohort also demonstrated cognitive impairment in PLWH with prior syphilis compared to PLWH without prior syphilis [6]. A recent study in a large cohort of individuals with acute HIV infection also showed poorer cognition in those with past or current syphilis [24]. In contrast, the POPPY study, which included older PLWH and controls without HIV, did not demonstrate a relationship between previous syphilis and cognitive function [16]. In the current study of individuals with active syphilis, we found that 40% experienced cognitive decline in the year after syphilis diagnosis, regardless of cognitive performance at entry. While other studies relied on different definitions of previous syphilis, included few individuals with active syphilis, and were cross sectional, our study participants had active syphilis and were followed with serial standardized assessments for one year.

Our finding that CSF pleocytosis in individuals with syphilis increased the odds of more severe cognitive impairment is in keeping with our understanding of the neuropathogenesis of syphilis. Individuals with cognitive impairment accompanied by CSF pleocytosis meet an accepted case definition of symptomatic neurosyphilis [5], and we would expect them to improve with neurosyphilis therapy. On the other hand, individuals with cognitive impairment but normal CSF WBC do not meet the case definition, and based on CSF findings, don’t merit neurosyphilis therapy. We found that change in cognition, be it normalization, improvement or worsening, did not differ between participants who did not undergo LP and those who did undergo LP and were treated based on CSF findings. These findings might be interpreted as indicating that treatment based on CSF findings does not impact syphilis outcome. However, participants who underwent LP and were treated for neurosyphilis or not, according to CSF findings, might define a more rigorous comparison. When examining only those whose CSF WBC count was determined, we found that individuals with CSF pleocytosis treated for neurosyphilis were less likely to experience cognitive decline in the year following treatment than those without CSF pleocytosis who were not treated. This suggests that treatment based on CSF findings did improve outcome in these individuals.

Previous work has shown that individuals with syphilis who do not have neurosyphilis may experience cognitive impairment [6, 7, 24]. Individuals with syphilis mount a robust peripheral humoral, cellular and inflammatory cytokine response [28–30] which, even without direct CNS infection, could increase vulnerability to cognitive decline. While not proven in the setting of syphilis, the role of systemic inflammation in cognitive decline is demonstrated in Alzheimer’s disease [31]. Perhaps more relevant to our study participants, peripheral systemic inflammation in mid-life predicts cognitive decline over time [32, 33]. We hypothesize that individuals with neurosyphilis and CSF inflammation experience an additional cognitive insult that amplifies that caused by peripheral *T*. *pallidum* infection. Treatment of those with CSF inflammation may mitigate this second insult or at least attenuate cognitive decline.

We acknowledge limitations of our study. Inclusion of a non-randomized group introduces risk of bias; however, the groups were comparable with regard to neurosyphilis risk factors and taking randomization status into account in our analyses did not alter our findings. We did not administer a detailed assessment of cognitive complaints. However, previous work in PLWH [25, 34, 35] and in others with memory complaints [36, 37] shows that perceptions of cognition have poor validity compared to objective assessments [34, 35, 37]. We used normative data provided by CogState, which may or may not be pertinent to PLWH, although these have been used in other studies of PLWH [17]. Two of our randomized participants had been tested previously, but with at least a six-month interval between tests, which makes practice effects less likely. The number of individuals who underwent follow-up in each comparison group is small, limiting power.

Our findings have implications for clinical care of individuals with syphilis who are at high risk for laboratory-defined neurosyphilis. Cognitive complaints were common but did not correlate with abnormalities on the CogState, suggesting that objective assessments are required to identify individuals with syphilis who have cognitive impairment. We also show that severity of cognitive impairment is greater in those who have CSF pleocytosis, and that identification and treatment of these individuals for neurosyphilis may mitigate subsequent cognitive decline. Our findings argue that the debate over LP is not yet resolved for patients with syphilis who have high serum RPR titers, or in PLWH, who have low CD4+T cells.

## Supporting information

S1 Table Relationship between cognitive complaints and performance on CogState and cerebrospinal fluid abnormalities. (DOCX)

S2 Table Factors that did not influence severity of cognitive impairment as assessed by CogState. (DOCX)

S3 Table Adjusted odds ratios of factors that influence severity of cognitive impairment as assessed by CogState, including randomization status. (DOCX)

S4 Table Hazard ratios for normalization or improvement in performance on the CogState battery if treatment based on CSF findings vs. treatment not based on CSF findings. (DOCX)
