# Supplementary material for: Cognitive impairment in syphilis: Does treatment based on cerebrospinal fluid analysis improve outcome?
Source: PLoS One. 2021 Jul 13;16(7):e0254518. doi: 10.1371/journal.pone.0254518 (PMC8277035; doi:10.1371/journal.pone.0254518)
Supplement: S1 Table — (DOCX) [file pone.0254518.s001.docx]

**S1 Table. Relationship between cognitive complaints and performance on CogState and cerebrospinal fluid abnormalities**

|  | No cognitive complaints | Cognitive Complaints | P-value |
| --- | --- | --- | --- |
| Abnormal performance on CogState | 28/60 (46.7)% | 32/60 (53.3%) | 0.26 |
| CSF WBC >5/uL | 13/30 (43.3%) | 17/30 (56.7%) | 0.52 |
| Reactive CSF-VDRL | 3/4 (75.0%) | 1/4 (25.0%) | 0.34 |

Cognitive complaints were assessed by Beck Depression Inventory-II or Lawton and Brody instrumental activities of daily living scale.

CSF, cerebrospinal fluid; VDRL, Venereal Disease Research Laboratory test; WBC, white blood cells
