# Supplementary material for: Cognitive impairment in syphilis: Does treatment based on cerebrospinal fluid analysis improve outcome?
Source: PLoS One. 2021 Jul 13;16(7):e0254518. doi: 10.1371/journal.pone.0254518 (PMC8277035; doi:10.1371/journal.pone.0254518)
Supplement: S2 Table — (DOCX) [file pone.0254518.s002.docx]

**S2 Table. Factors that did not influence severity of cognitive impairment as assessed by CogState**

|  | Severity of Cognitive Impairment |
| --- | --- |
| Factor | OR (95% CI), p=value |
| Randomized | 0.7 (0.3-1.6), p=0.43 |
| PLWH | 1.0 (0.5-2.2), p=0.98 |
| Treatment of the current episode of syphilis prior to study entry | 0.6 (0.2-1.3), p=0.20 |
| Serum RPR titer (per 2-fold increase in titer) | 1.0 (0.8-1.2), p=0.83 |
| Syphilis stage (early vs. late) | 0.8 (0.3-2.2), p=0.62 |
| Detection of *T. pallidum* DNA in blood | 2.7 (0.7-10.2), p=0.15 |
| Prior syphilis | 1.1 (0.5-2.4), p=0.79 |
| Urine toxicology screen positive for amphetamine/methamphetamine | 1.0 (0.5-2.4), p=0.93 |
| Urine toxicology screen positive for THC | 0.8 (0.3-1.8), p=0.54 |
