# Supplementary material for: Cognitive impairment in syphilis: Does treatment based on cerebrospinal fluid analysis improve outcome?
Source: PLoS One. 2021 Jul 13;16(7):e0254518. doi: 10.1371/journal.pone.0254518 (PMC8277035; doi:10.1371/journal.pone.0254518)
Supplement: S3 Table — (DOCX) [file pone.0254518.s003.docx]

**S3 Table. Adjusted Odds Ratios of factors that influence severity of cognitive impairment as assessed by CogState, including randomization status**

|  | Severity of Cognitive Impairment |
| --- | --- |
| Factor | aOR (95% CI), p-value |
| CSF WBCs > 5/ul | 4.9 (1.7-14.4), p=0.004 |
| Total BDI-II ≥14 | 6.0 (1.9-18.8), p=0.002 |
| ≤ 12 years of education | 3.3 (1.0-10.5), p=0.05 |
| Randomized | 0.3 (0.1-1.0), p=0.05 |
