# Supplementary material for: Cognitive impairment in syphilis: Does treatment based on cerebrospinal fluid analysis improve outcome?
Source: PLoS One. 2021 Jul 13;16(7):e0254518. doi: 10.1371/journal.pone.0254518 (PMC8277035; doi:10.1371/journal.pone.0254518)
Supplement: S4 Table — (DOCX) [file pone.0254518.s004.docx]

**S4 Table. Hazard ratios for normalization or improvement in performance on the CogState battery if treatment based on CSF findings vs. treatment not based on CSF findings**

|  | Hazard Ratio (95% CI), p-value |
| --- | --- |
| Improvement in CogState | 0.8 (0.3-2.0), p=0.69 |
| Normalization of CogState | 0.7 (0.1-3.2), p=0.59 |
